# Supplementary material for: Feasibility of Estimation of Aortic Wave Intensity Using Non-invasive Pressure Recordings in the Absence of Flow Velocity in Man
Source: Front Physiol. 2020 May 25;11:550. doi: 10.3389/fphys.2020.00550 (PMC7260344; doi:10.3389/fphys.2020.00550)
Supplement: Supplementary file 1 [file Data_Sheet_1.PDF]

Supplementary figures

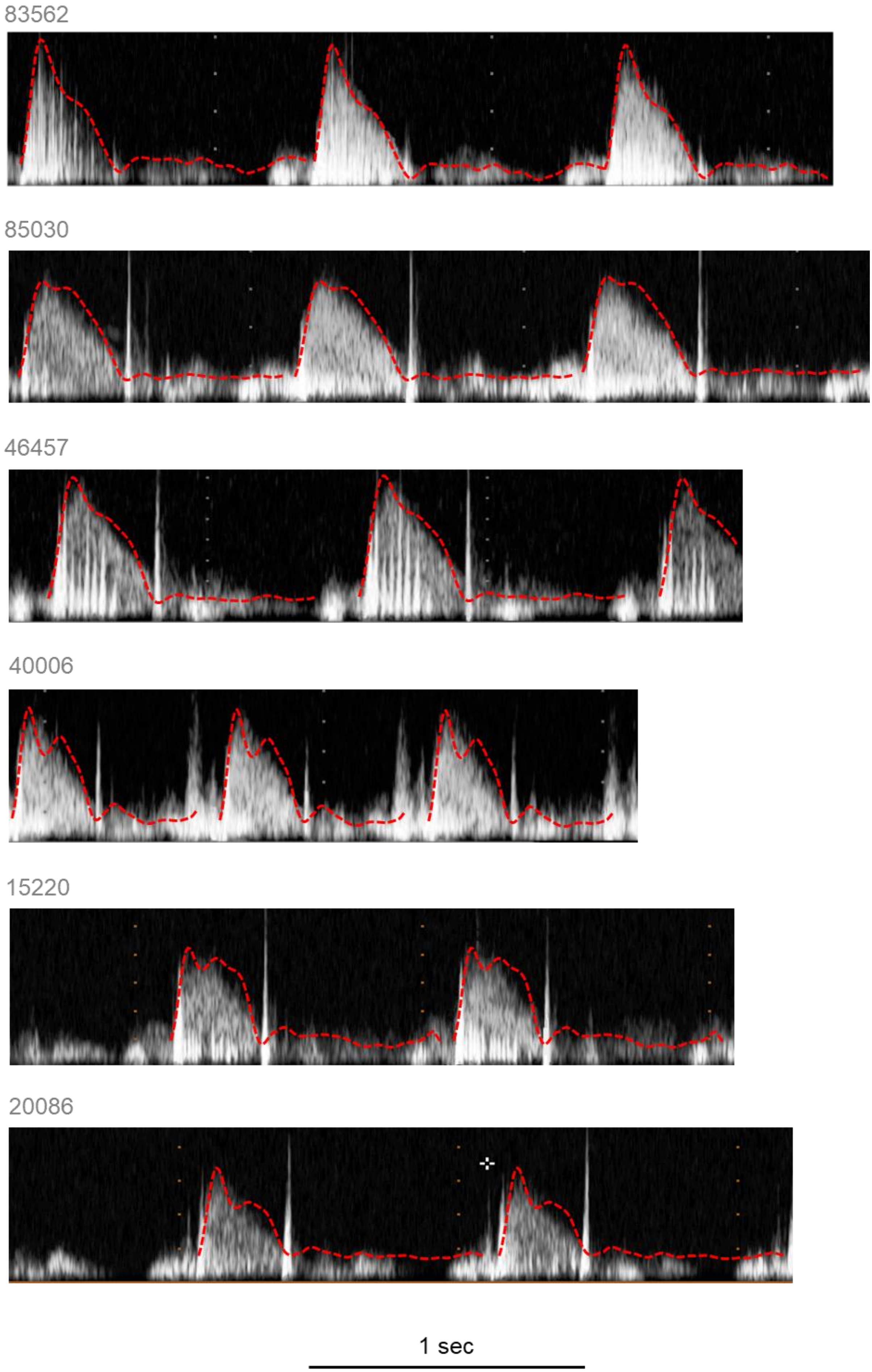
83562
85030
46457
40006
15220
20086
1 sec

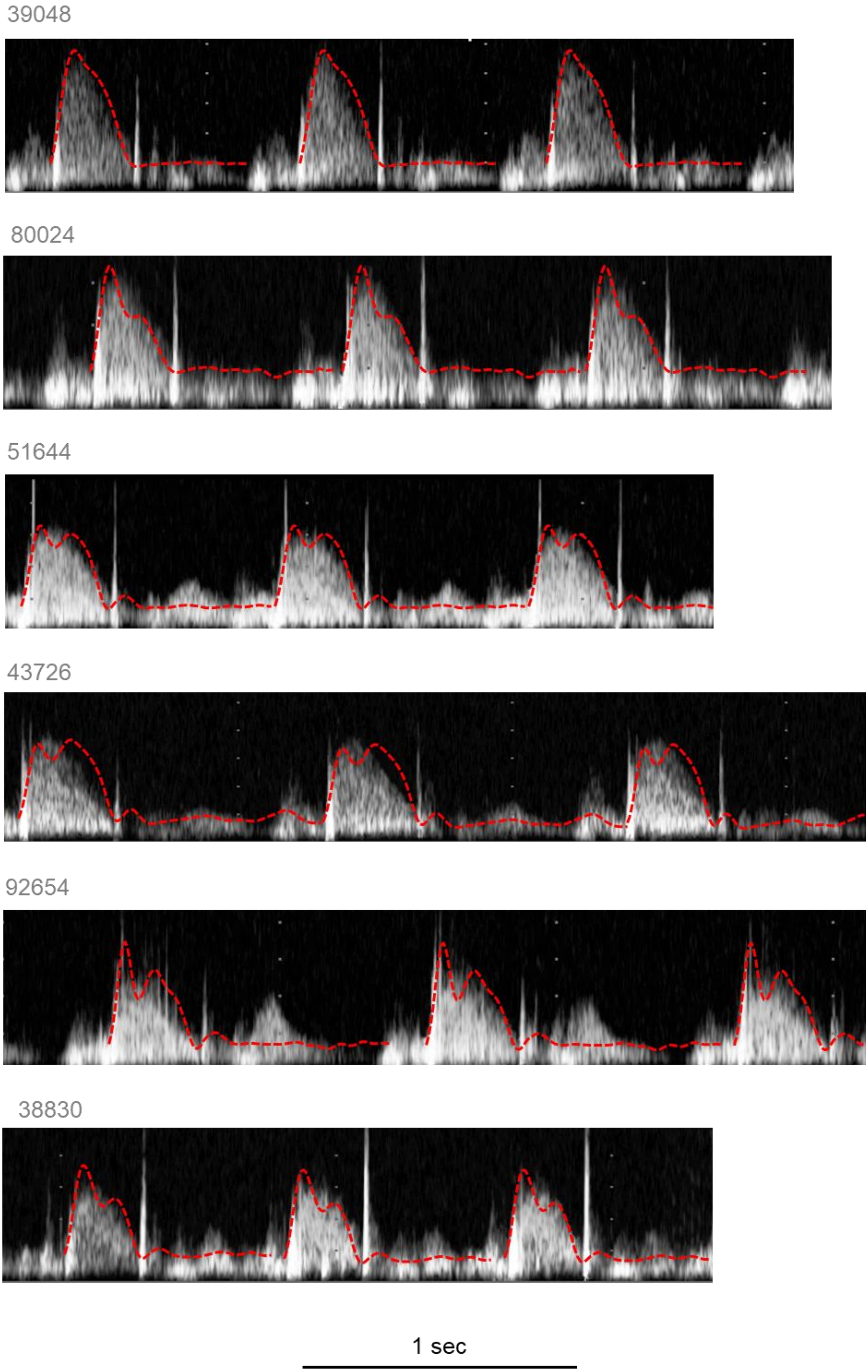
39048
80024
51644
43726
92654
38830
1 sec

Figure S1. Velocity traces from the left ventricular outflow tract with the respective P_{xs} derived from the central (aortic) waveform (red dashes) are shown. Waveforms were scaled to correspond with the peak of the aortic flow waveform.

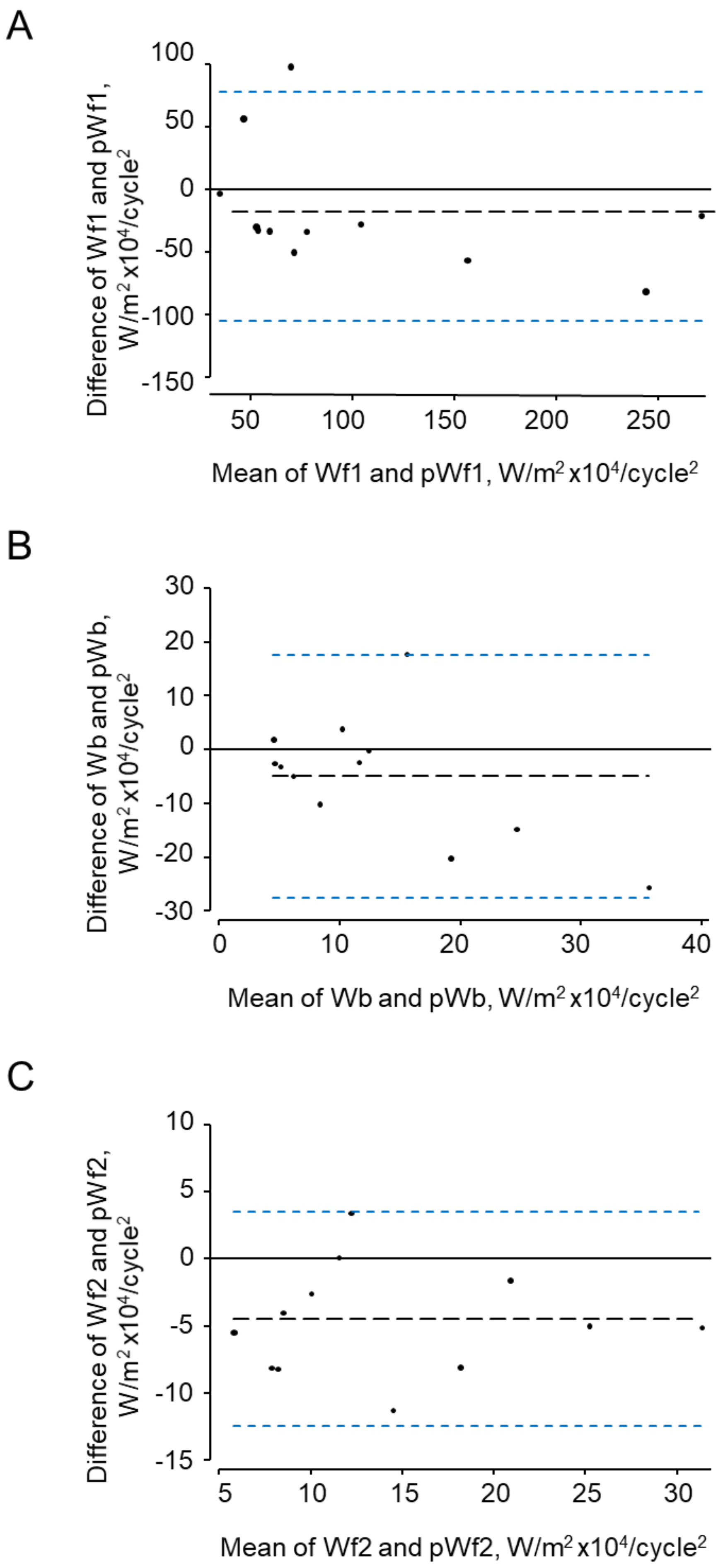
A
Difference of Wf1 and pWf1, W/m2 x104/cycle2
Mean of Wf1 and pWf1, W/m2 x104/cycle2
B
Difference of Wb and pWb, W/m2 x104/cycle2
Mean of Wb and pWb, W/m2 x104/cycle2
C
Difference of Wf2 and pWf2, W/m2 x104/cycle2
Mean of Wf2 and pWf2, W/m2 x104/cycle2

Figure S2. Bland-Altman plots showing agreement between the three major waves identified by traditional (Wf1, Wb, Wf2) and pressure-only wave intensity analysis (pWf1, pWb, pWf2) calibrated to a constant peak aortic velocity of 1m/s for all traces. (mean difference Wf1 = -18 (LOA -115, 79) $W/m^2 \times 10^4/cycle^2$, rho = 0.80; Wb = -50 (LOA -28, 18) $W/m^2 \times 10^4/cycle^2$, rho = 0.41; -47 (LOA -13, 33) $W/m^2 \times 10^4/cycle^2$, rho = 0.74).
